# Light-activatable and hyperthermia-sensitive “all-in-one” theranostics: NIR-II fluorescence imaging and chemo-photothermal therapy of subcutaneous glioblastoma by temperature-sensitive liposome-containing AIEgens and paclitaxel

**DOI:** 10.3389/fbioe.2023.1343694

**Published:** 2023-12-28

**Authors:** Lixin Du, Pan Wang, Haiyan Huang, Menglong Li, Shubham Roy, Yinghe Zhang, Bing Guo

**Affiliations:** ^1^ Department of Medical Imaging, Shenzhen Longhua District Central Hospital, The Key Laboratory of Neuroimaging, Shenzhen, China; ^2^ School of Science, Shenzhen Key Laboratory of Flexible Printed Electronics Technology, Shenzhen Key Laboratory of Advanced Functional Carbon Materials Research and Comprehensive Application, Harbin Institute of Technology, Shenzhen, China

**Keywords:** temperature-sensitive liposomes, glioblastoma, combinatory photothermal and chemotherapy, NIR-II fluorescence imaging, nanomedicines

## Abstract

Nowadays, it is still quite difficult to combat glioblastoma, which is one of the most lethal cancers for human beings. Combinatory therapy, which could not only improve therapeutic efficacy and overcome multiple drug resistance but also decrease the threshold therapeutic drug dosage and minimize side effects, would be an appealing candidate for glioblastoma treatment. Herein, we report fluorescence imaging in the second near-infrared window (NIR-II)-guided combinatory photothermal therapy (PTT) and chemotherapy of glioblastoma with a newly formulated nanomedicine termed **PATSL**. It is composed of temperature-sensitive liposome (TSL) carriers, NIR-II emissive and photothermal aggregation-induced emission (AIE) dyes, and chemotherapeutic paclitaxel (PTX) as well. **PATSL** shows spherical morphology with diameters of approximately 55 and 85 nm by transmission electron microscopy and laser light scattering, respectively, a zeta potential of −14.83 mV, good stability in both size and photoactivity, strong light absorption with a peak of approximately 770 nm, and bright emission from 900 nm to 1,200 nm. After excitation with an 808-nm laser with good spatiotemporal controllability, **PATSL** emits bright NIR-II fluorescence signals for tumor diagnosis *in vivo*, exhibits high photothermal conversion efficiency (68.8%), and triggers drug release of PTX under hypothermia, which assists in efficient tumor ablation *in vitro* and *in vivo*. This research demonstrates that “all-in-one” theranostics with NIR-II fluorescence imaging-guided combinatory PTT and chemotherapy is an efficient treatment paradigm for improving the prognosis of brain cancers.

## 1 Introduction

Nowadays, it is still quite challenging to treat glioblastoma with conventional therapeutic paradigms like surgery and postoperative chemotherapy, leading to the low median life span (<2 years) for diagnosed patients (Omuro and DeAngelis, 2013; Grauwet and Chiocca, 2016; Aldape et al., 2019; Sampson et al., 2020; Qiao et al., 2022). The most probable reasons include (i) the existence of the blood–brain barrier hinders drug penetration and minimizes the efficacy of diagnosis and therapeutics; (ii) the surgery could not clear the tiny lesion tissues, which naturally prefer to penetrate deep of the central nerve system and this would lead to tumor recurrence; and (iii) while chemotherapy is the most often used therapeutic modality, the tumor gradually exhibits multidrug resistance under repeated treatment of drugs with high dosage, and the strong side effects of chemotherapy also severely compromise the health of patients (Sheng et al., 2018; Tang et al., 2019a; Agrawala et al., 2020; Bastiancich et al., 2021; Quader et al., 2022).

Photothermal therapy (PTT) as an emerging therapeutic modality relies on photothermal agents to convert spatiotemporal controllable light energy to hyperthermia *via* a non-radiative decay pathway for excitons (Guo et al., 2017; Guo et al., 2018; Zhang et al., 2022a). Importantly, PTT is a light-activatable precision therapy with minimal invasiveness and low side effects to locally ablate tumor tissues, holding great promise in cancer therapy. However, the light penetration depth for even near-infrared lasers is still less than 1 cm, in which it is difficult for hyperthermia to approach the infiltrating tumor tissues beneath deep tissue (Luo et al., 2016; Guo et al., 2018; Hu et al., 2022a). Although it is possible to increase the light penetration of the laser source with high power density to ablate the infiltrating tumors, overheating during the photothermal treatment would cause unwanted side effects for surrounding health tissues (Hu et al., 2022b). Thus, it is ideal to take advantage of the merits of PTT, while overcoming its drawbacks.

So far, photothermal agents reported in literature are generally composed of organic materials and/or inorganic materials (Cai et al., 2016; Guo et al., 2016; Lu et al., 2022; Cui et al., 2023). For inorganic materials, there is a concern for their long-term safety (Guo et al., 2016). For organic materials, they generally include conjugated polymers and small organic molecules. It is noticed that conjugated polymers often suffer from low solubility, making it difficult to process them into water-dispersible nanoparticles, and low reproducibility, because of their intrinsic and rather high and wide molecular weight distribution (Lu et al., 2022). In contrast, small organic molecules with precise molecular structure show good reproducibility and ease in tuning photophysical properties by following a molecular engineering approach, which is appealing for the nanomedicine industry (Cai et al., 2016). Among different photothermal small organic molecules, aggregation-induced emission (AIE) luminophores with absorption in the first near-infrared (NIR-I) window not only show photothermal effects but also often demonstrate bright emission in the second NIR (NIR-II) window (Gao et al., 2019; Liu et al., 2019; Jiang et al., 2021; Li et al., 2021; Yan et al., 2021). It has been demonstrated that AIE luminophores are good candidates of “all-in-one” theranostics for NIR-II fluorescence imaging-guided PTT. Notably, rather than conventional NIR-I fluorescence imaging used in the clinic, NIR-II fluorescence imaging is burgeoning for clinic translation because of its superiority in deep penetration, a high signal-to-background ratio, and good spatiotemporal resolution (Tang et al., 2019b; Li et al., 2020; Liu et al., 2022; Chen et al., 2023).

So far, chemotherapy as the most often used therapeutic modality for cancer treatment shows non-limited penetration depth, which could be effective at the tumor margin where hyperthermia cannot reach (Zhang et al., 2022b). Importantly, combinatory therapy integrated with different therapeutics could inherit the pros of the corresponding therapeutic modalities, decrease the therapeutic threshold drug dosage, minimize the side effects, overcome multidrug resistance, and improve the prognosis for patients after treatment (Fan et al., 2017; Guo et al., 2020). Therefore, it is expected that nanomedicines, which contain photothermal and chemotherapeutic drugs, could not only exhibit the advantages of conventional combinatory therapy but also show a synergistic effect. This synergistic effect is due to that the localized hyperthermia generated by photothermal treatment could rupture the drug carrier, trigger drug release, temporally disrupt the cell membrane, overcome the obstacles of tumor physiology, improve permeability of the drug in the targeted tissue, and facilitate nanomedicine transportation into tumor cells, leading to high localized drug concentration to effectively ablate tumors (Ribeiro et al., 2022; Wu et al., 2022). Therefore, the combinatory PTT and chemotherapy could achieve good treatment outcomes using medium photothermal laser power to prevent overheating effects, minimize unwanted damage to surrounding normal tissues, decrease therapeutic threshold dosage of chemodrugs, and avoid severe side effects because of the cytotoxicity induced by the drug released during circulation in blood.

**SCHEME 1:**
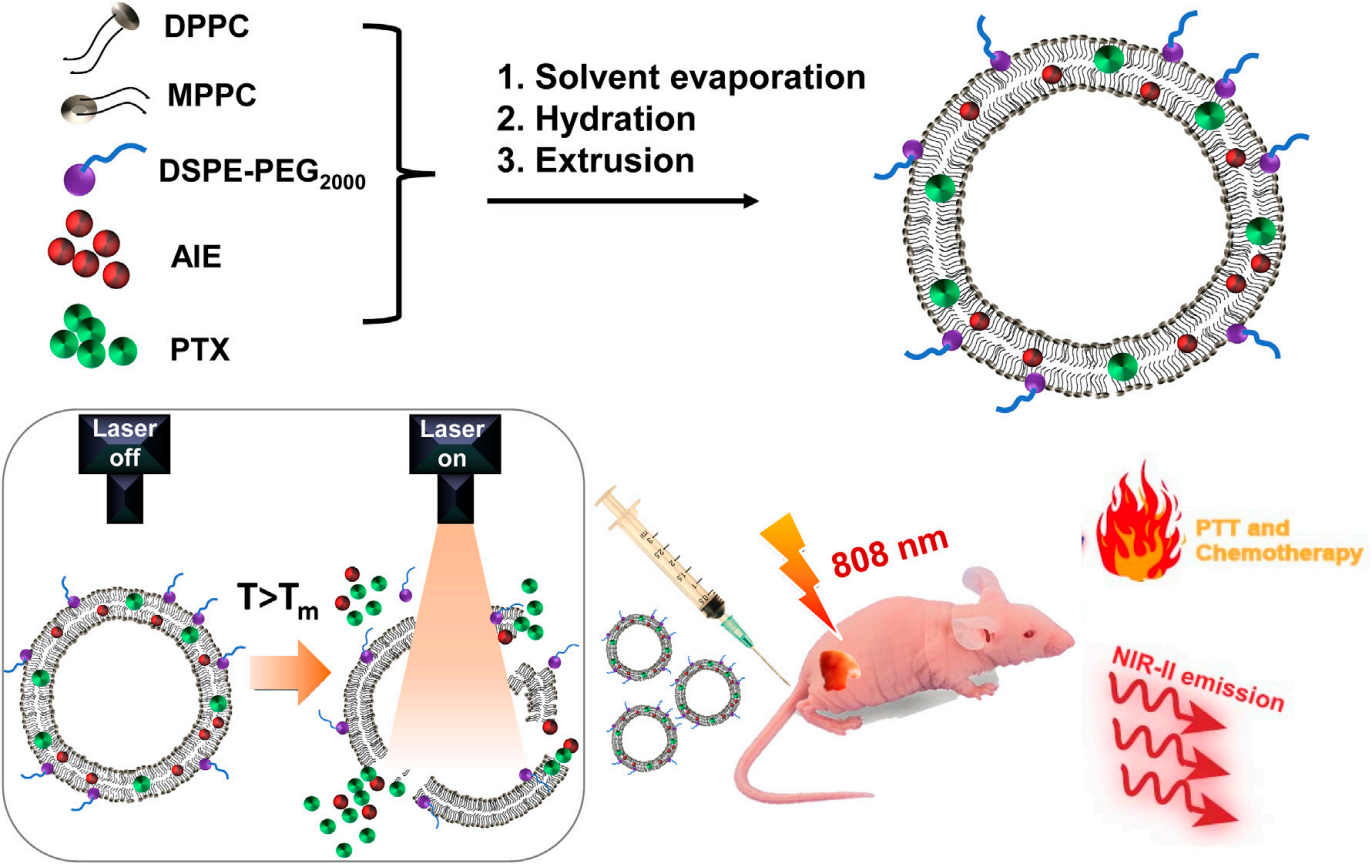
Illustration of the formulation of light-activatable and hyperthermia-sensitive “all-in-one” theranostic nanomedicine **PATSL** and the application in NIR-II fluorescence imaging and chemo-photothermal therapy of subcutaneous glioblastoma by temperature-sensitive liposome-containing AIEgens and paclitaxel.

For drug delivery, nanocarriers usually contribute to controlled release with minimized drug release during circulation and low side effects and efficient accumulation in cancer tissues for therapy (Bhatia et al., 2021). The emerging stimulus nanocarriers could generate on-demand release of drugs, which is even superior to conventional nanocarriers (Song et al., 2022). As one of the presentative stimulus nanocarriers, temperature-sensitive liposomes (TSLs) not only could encapsulate both hydrophobic and hydrophilic drugs but also show triggered drug release under hyperthermia, leading to boosted drug accumulation in tumor tissues (Deng et al., 2016; Yuba et al., 2021; Fu et al., 2023). This is beneficial to lower down the therapeutic threshold drug dosage and achieve minimized unwanted drug release during circulation with low side effects. Therefore, TSLs are excellent candidates for combinatory PTT and chemotherapy. Importantly, the light-activatable photothermal agents loaded in TSLs could contribute to on-demand release of drugs from the liposome carrier under laser irradiation with spatiotemporal controllability (Cheng et al., 2021; Peng et al., 2023). In this contribution, we used a TSL carrier to deliver NIR-II AIE dye **TB1** and paclitaxel (PTX) with production of “all-in-one” theranostic nanomedicines (**PATSL**) for light-activatable NIR-II fluorescence imaging-guided combinatory on-demand PTT and chemotherapy of glioblastoma. We started from the nanomedicine formulation, followed by systematic characterization of their photophysical properties, morphology testing, study of drug release behavior under hyperthermia, and photothermal effect investigation. More importantly, the diagnosis and inhibition of the tumor with **PATSL** were carefully examined both *in vitro* and *in vivo* (Scheme 1).

## 2 Results and discussion

In this study, the presentative NIR-II AIE dye **TB1** was formulated with a donor–acceptor engineering approach (Sheng et al., 2018), which contains benzobisthiadiazole core as the electron-deficient acceptor, conjugated with *N,N*-diphenyl- 4-(1,2,2-triphenylvinyl)aniline (DPTPEA) as the electron-rich donor. The rotary molecular structure makes the **TB1** molecules stack with each other in a solid state like in a hydrophobic layer of liposomes without fluorescence quenching, which is beneficial for bright NIR-II fluorescence imaging of tumors and real-time guidance of treatment *in vivo*. Furthermore, *via* a thin-film rehydration method, the TSL carrier composed of 1,2-dipalmitoyl-*sn*-glycero-3-phosphocholine (DPPC), 1-palmitoyl-2-hydroxy-sn-glycero-3-phosphocholine (MPPC), and 1,2 distearoyl-sn-glycero-3-phosphoethanolamine-*N*-methoxy polyethylene glycol-2000 (DSPE-PEG2000) was formulated to physically encapsulate **TB1** and the antitumor chemodrug PTX together in the phospholipid bilayer, yielding “all-in-one” theranostic nanomedicine **PATSL** for NIR-II fluorescence imaging-guided light-activatable and combinatory chemotherapy and PTT of subcutaneous glioblastoma (Scheme 1). Importantly, the presence of 808-nm light-activated TB1 molecules in the lipid bilayer spatiotemporally facilitates the dual NIR-II emission for imaging and hyperthermia, which contributes to PTT and disruption of the liposome shell as well, leading to burst release of PTX with high localized free drug concentration. Furthermore, the morphology characterization was conducted by both transmission electron microscopy (TEM) and dynamic light scattering (DLS). The results showed that the nanomedicine exhibits an average nanoparticle size of near 55 and 85 nm for TEM and DLS, respectively. In addition, the zeta potential was found to be approximately −14.83 mV because of PEGylation on the surface of liposomes. Moreover, the encapsulation efficiency of PTX in the nanomedicine was calculated to be approximately 65%, determined by high-performance liquid chromatography.

**FIGURE 1:**
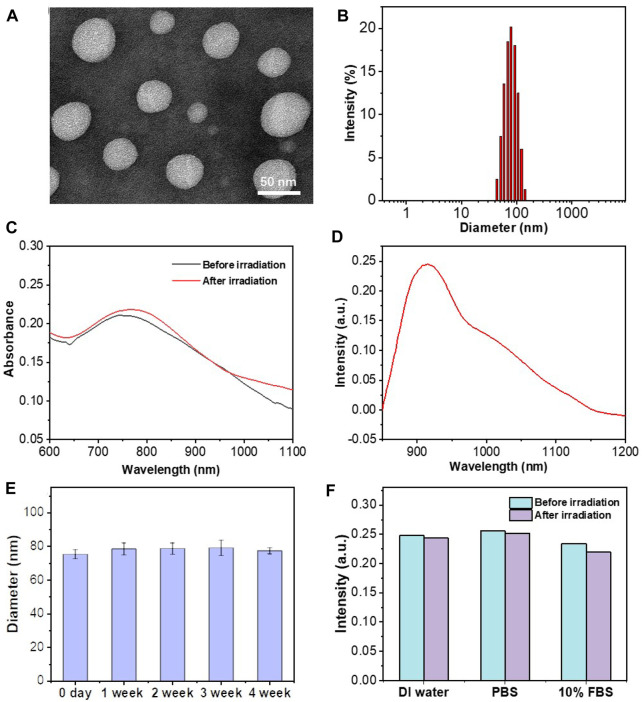
Morphology and photophysical characterization of liposomes. **(A)** TEM images of **PATSL**; **(B)** DLS result for **PATSL**. **(C)** UV–vis spectra of **PATSL** before and after NIR laser irradiation (808 nm, 0.8 W/cm^2^, 5 min). **(D)** PL spectrum of **PATSL** under 808 nm excitation. **(E)** Variation in the size of **PATSL** at different weeks. **(F)** PL spectrum of **PATSL** under laser irradiation (808 nm, 0.8 W/cm^2^, 5 min) in different media including DI water, PBS, and 10% FBS.

From the UV–vis absorption spectrum (Figure 1C), **PATSL** exhibited a strong NIR-II absorption peak at 760 nm in aqueous media. The strong NIR absorbance for the nanomedicine is owing to the intramolecular charge transfer effect (Sheng et al., 2018), which is the intrinsic nature of the conjugated donor–acceptor structured **TB1** molecules. Notably, the large extinction coefficient of **PATSL** would contribute to their strong photothermal effect under NIR laser irradiation. As shown in Figure 1D, the **PATSL** showed bright emission from 900 to 1,200 nm, which paves the way for their application in NIR-II fluorescence imaging *in vivo*. Importantly, the size of **PATSL** remained constant even after 4 weeks of storage under 4°C conditions (Figure 1E), which indicates that the nanomedicine could keep colloidal stability for a long time period. Furthermore, after continuous laser irradiation for 10 min, the UV–vis spectrum of **PATSL** in water did not show significant changes (Figure 1C), while the PL intensity even in different aqueous media did not show a notable decrease (Figure 1F). These results showed that **PATSL** have good photostability, which is appealing for repeated photothermal treatment and long-term fluorescence imaging *in vitro* and *in vivo*.

**FIGURE 2:**
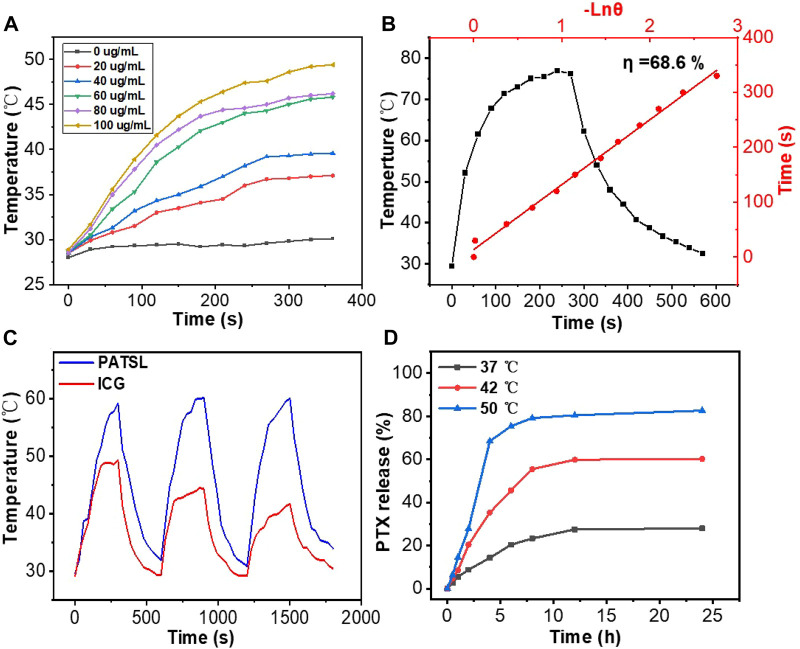
**(A)** Temperature change curves for **PATSL** in water with changing concentrations under continuous laser irradiation (808 nm, 0.8 W/cm^2^). **(B)** Temperature evolution data for **PATSL** (PTX + **TB1** concentration = 2 mg/mL, PTX:**TB1** = 1:1) under both NIR laser irradiation (808 nm, 0.8 W/cm^2^) and the cooling conditions. The time constant for heat transfer from the aqueous nanomedicine solution, which was calculated from the linear time data from the cooling period *versus* the negative natural logarithm of the system driving force temperature (Guo et al., 2016; Guo et al., 2018). **(C)** Cyclic photothermal heating and cooling of **PATSL** and ICG (dye concentration = 0.5 mg/mL). **(D)** Cumulative PTX release profile from the **PATSL** sample at 37°C, 42°C, or 50°C .

To examine the photothermal performance of the nanomedicine formulated, we monitored the temperature changes in aqueous **PATSL** at changing concentrations ranging from 0 to 100 μg/mL under continuous laser irradiation (808 nm, 0.8 W/cm^2^). As shown in Figure 2A, the temperature of the samples increased faster at the higher concentration of aqueous **PATSL** with increasing irradiation time. For instance, the hyperthermia effect of **PATSL** was notably evident, displaying a rapid temperature increase from room temperature to 49.4°C upon laser irradiation for 6 min, in which the hyperthermia effect would be sufficient to ablate cancers *in vitro* and *in vivo*. In contrast, the temperature of pure water only increased from 27.9°C to 29.9°C under the same conditions. These results suggest that aqueous **PATSL** could efficiently convert light energy to heat energy through the non-radiative decay pathway for the exciton of **TB1** under laser irradiation (Cai et al., 2016; Guo et al., 2016; Guo et al., 2017; Guo et al., 2018; Gao et al., 2019; Liu et al., 2019; Jiang et al., 2021; Li et al., 2021; Yan et al., 2021; Hu et al., 2022a; Zhang et al., 2022a; Lu et al., 2022; Cui et al., 2023). Notably, the quantitative photothermal conversion efficiency was calculated to be approximately 68.6% (Figure 2B). (Guo et al., 2016; Guo et al., 2018) The cyclic photothermal heating and cooling processes were further conducted *in vitro*, and the results showed that the nanomedicines could be reversibly heated under laser irradiation for three cycles without significant changes in their hyperthermia properties. In contrast, indocyanine green (ICG), as a presentative commercial NIR-II fluorescent and photothermal dye showed obvious decay in the photothermal conversion cycles under laser irradiation at the same conditions for aqueous **PATSL** (Figure 2C). These results suggest that the aqueous **PATSL** have good photostability, which is consistent with the results in Figure 1C.

To study the temperature-sensitive drug release performance, aqueous **PATSL** samples were heated at hyperthermia temperatures including 42°C and 50°C and at body temperature (37°C) (Figure 2D). It is observed that PTX was released quickly, with 68% and 36% release at 4 min upon heating at 50°C and 42°C, respectively. In the 37°C condition, only 13% release of PTX was detected, which was much lower than that at the higher temperature. These results suggest that the nanomedicine has good temperature-sensitive performance with sharply increased drug release under the condition of the evaluated temperature.

**FIGURE 3:**
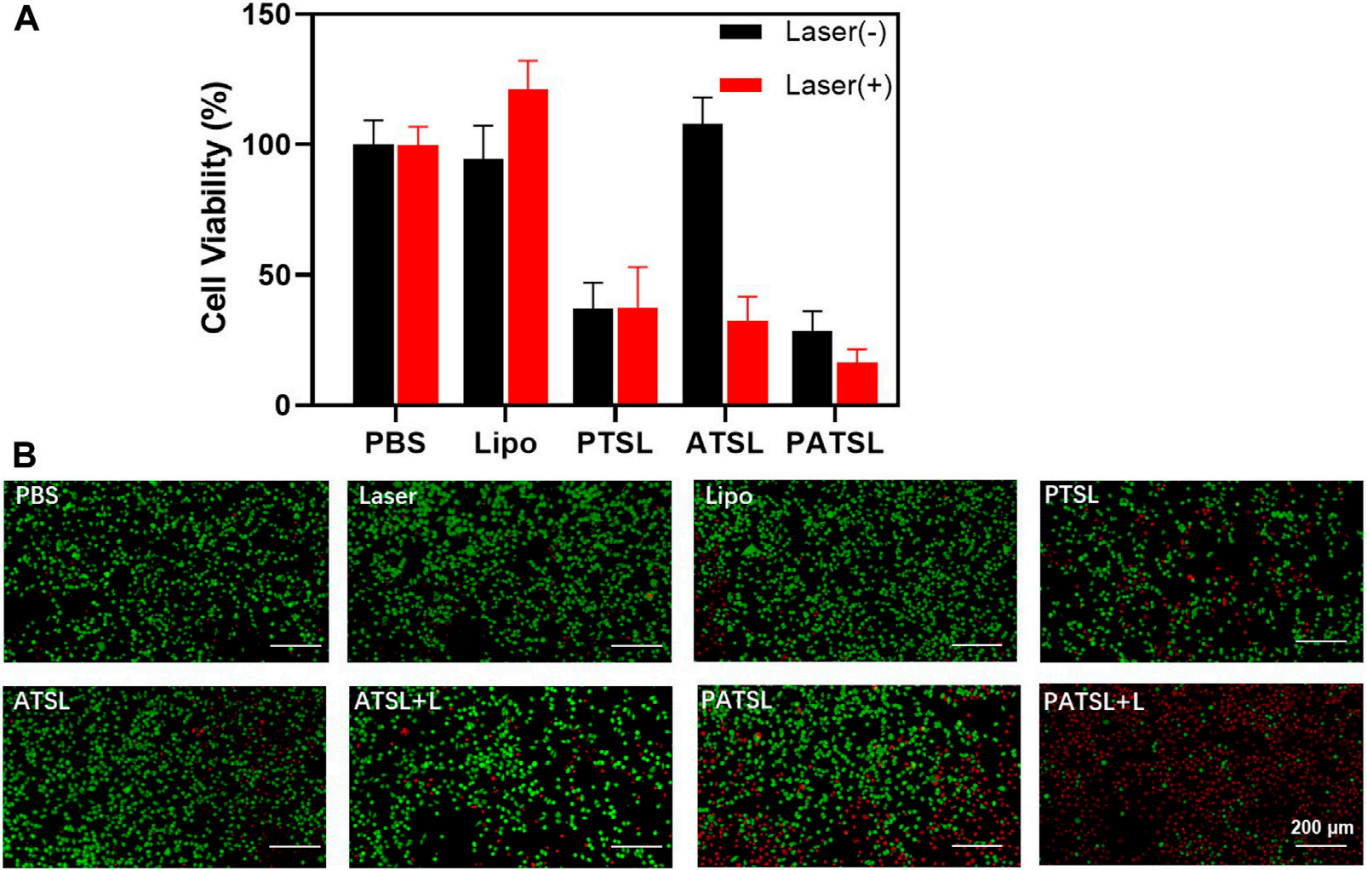
Cytotoxicity evaluation of nanomedicines. **(A)** U87 glioma cell viability for treatment of different groups including PBS, Lipo, PTSL, ATSL, and **PATSL** with and without laser irradiation. **(B)** Fluorescence changes in live and/or dead U87 glioma cells treated with 0.015 mg/mL under continuous NIR laser irradiation (808 nm, 0.8 W/cm^2^, 5 min). Calcein-AM indicator (with green emission) and propidium iodide indicator (with red emission) were applied to stain live and dead U87 glioma cells, respectively.

Before the *in vivo* study, we conducted an *in vitro* investigation of the chemotherapy, PTT, and light-activatable combinatory photothermal and chemotherapeutic efficacy, and the standard CCK-8 assay results are shown in Figure 3. It was found that the TSL carrier itself, **TB1** molecules, and laser irradiation do not cause obvious harm to cell growth, suggesting relatively good biocompatibility. Furthermore, the singular chemotherapy and PTT could obviously inhibit cell viability. More importantly, the **PATSL** group with dual **TB1** and PTX drug under laser irradiation showed higher cytotoxicity than the singular PTT and chemotherapy groups (ATSL + Laser and **PATSL**), respectively. To vividly evaluate the cytotoxicity of the different groups, green-emissive calcein-AM and red-emissive propidium iodide were taken to conduct the live/dead staining experiment, in which they could effectively stain live and dead cells, respectively (Figure 3B). The results for the control group of (laser, Lipo, and ATSL) showed that bright green fluorescence suggests that the laser condition does not significantly impact cell health, while the nanomedicine carrier and the **TB1** molecules do no obvious harm for cellular survival. Taken together, these results indicate that **PATSL** holds great promise for *in vivo* treatment of gliomas with light-activatable dual PTT and chemotherapy.

**FIGURE 4:**
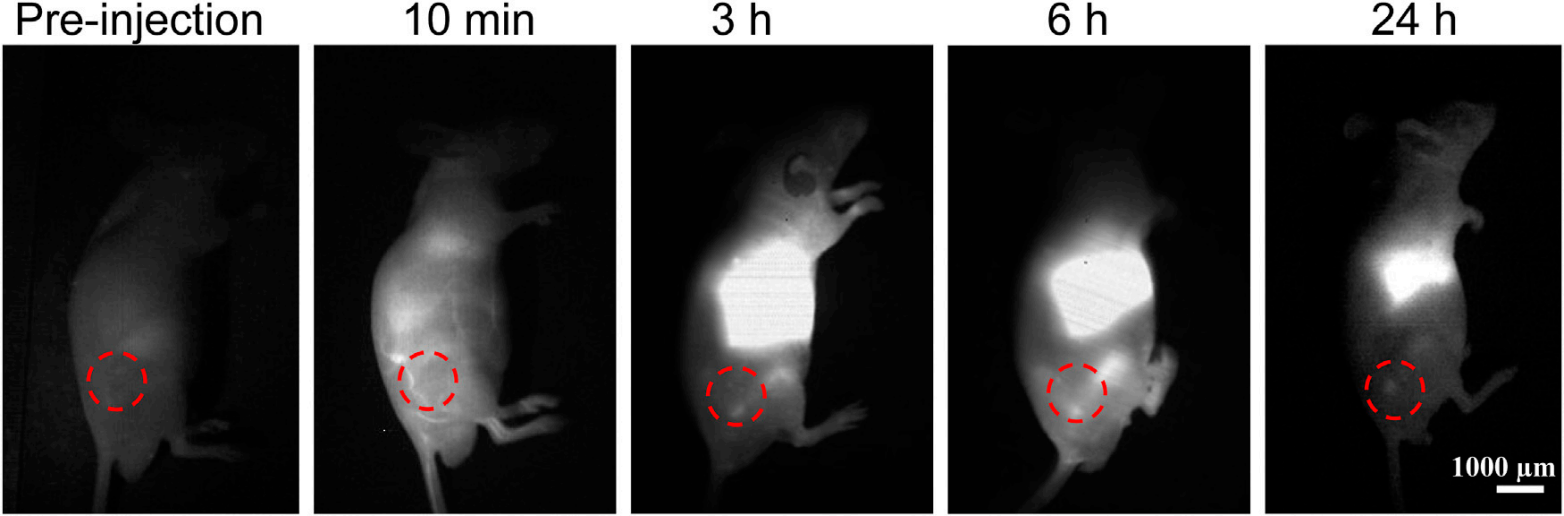
*In vivo* NIR-II fluorescence imaging (1000 LP, 100 ms) of mice bearing subcutaneous glioma under continuous 808 nm laser illumination with a power density of 60 mW/cm^2^.

For the study of the *in vivo* imaging of nanomedicine accumulation in tumors, we constructed a subcutaneous glioma model (Figure 4). The InGaAs camera was used to continuously capture the NIR-II fluorescence imaging signals with a long pass filter of 1,000 nm and exposure time of 100 ms on mice bearing subcutaneous glioma under NIR laser irradiation (808 nm, 60 mW/cm^2^). According to the experimental results, it was found that there were minimal NIR-II fluorescence signals for mice before **PATSL** injection. Upon systemic administration of **PATSL** (0.5 mg/Kg) *via* the tail vein, the strong fluorescence signals were detected on the mice’s body, especially in blood vessels. With increase in the post-injection time, the signals in the liver and tumor gradually increased and reached to a plateau at 6 h post-injection, indicating that most of the **PATSL** nanomedicine was captured in the liver and accumulated in the tumor as well. It is also noticed that after 24 h post-injection, the fluorescence signals in blood vessels diminished greatly and the tumor/liver fluorescence signals also decreased sharply, suggesting that nanomedicines were gradually cleared in the tumor/liver afterward.

**FIGURE 5:**
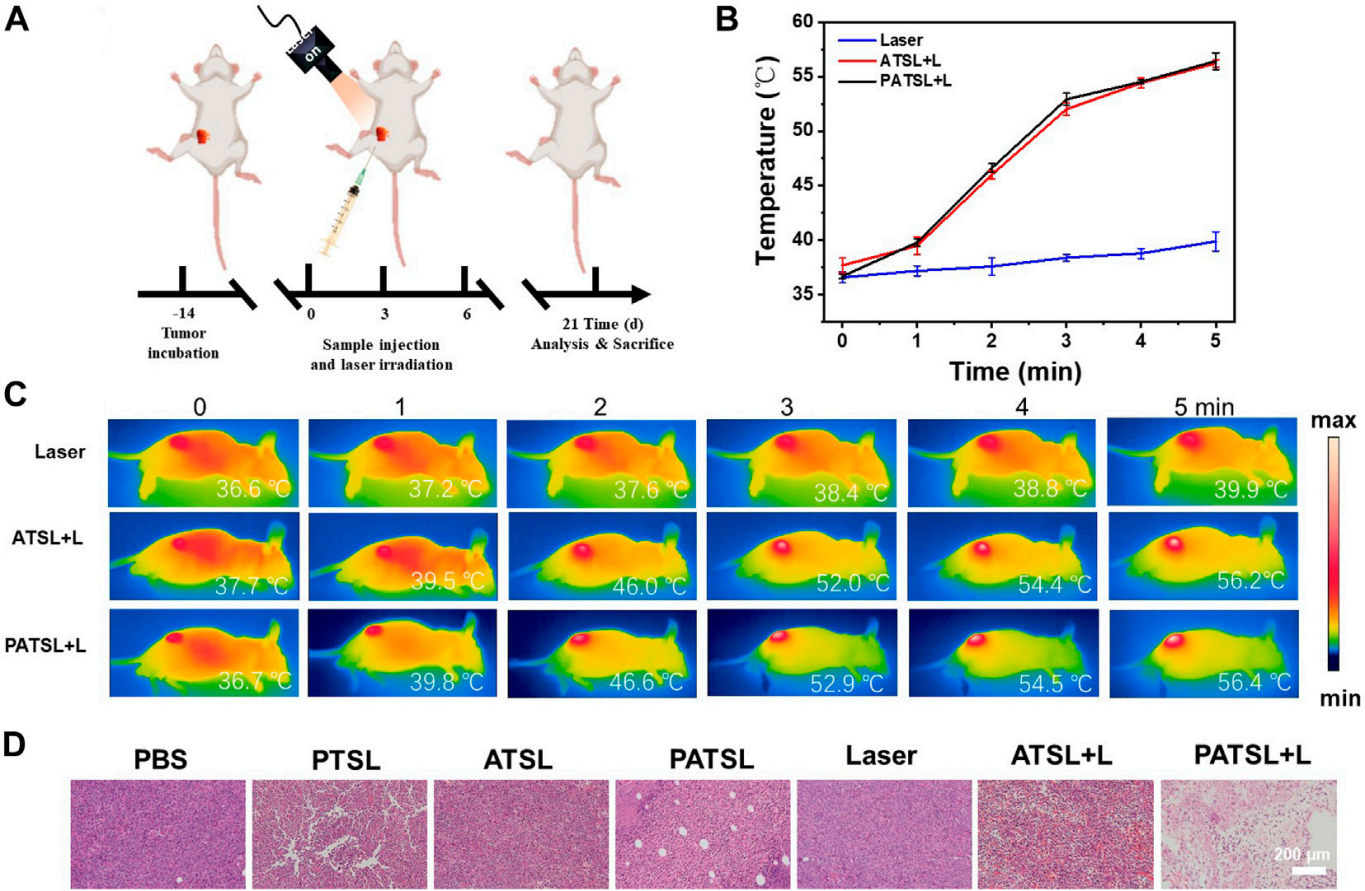
*In vivo* antitumor evaluation with and without NIR irradiation (808 nm, 0.8 W cm^-2^, 5 min) for subcutaneous glioma tumor-bearing mice. **(A)** Schematic illustration for tumor model establishment and the conduction process for the therapeutic treatment. **(B)** Temperature curves of the tumor tissue upon laser irradiation. **(C)** Infrared photos for mice under photothermal treatment under NIR irradiation (808 nm, 0.8 W cm^-2^, 5 min), and **(D)** H&E stained images of tumor sections of mice after 4 h of therapeutic treatment for different groups, which include (i) PBS, (ii) PTSL, (iii) ATSL, (iv) PATS, (v) Laser, (vi) ATSL + L, and (vii) **PATSL** + L.

To evaluate the photothermal and synergistic chemo- and photothermal capability *in vivo*, **ATSL** (**TB1**, 1 mg kg^-1^) and **PATSL** (PTX + **TB1**, 2 mg kg^-1^, PTX:**TB1** = 1: 1) samples were intratumorally injected into the mice, respectively. All of the three groups (Laser, **ATSL,** and **PATSL** groups) were irradiated with 808 nm laser (0.8 W/cm^2^, 5 min) at 6 h post-injection. Importantly, an infrared camera was used to continuously monitor the temperature changes in the mice in the experiment during photothermal treatment (Figure 5). It was found that the average temperature of the mice in the “control” group only increased from body temperature to 39.9°C within 5 min, while the average temperatures of mice in the **ATSL** and **PATSL** groups increased to near 56.3°C within 5 min. This suggests the good photothermal conversion capability of **TB1**-containing liposome nanomedicines *in vivo*. Notably, the local hyperthermia led not only to tumor cell death but also triggered the release of PTX to exert synergistic dual PTT and chemotherapy. To primarily verify the efficacy of chemotherapy, PTT, and synergistic PTT and chemotherapy in this study, hematoxylin and eosin (H&E) staining analysis was conducted after different treatments (Figure 5D). The results suggest that (i) cell necrosis plays a major role in damaging tumor tissues by local hyperthermia during PTT treatment; (ii) neither nanomedicines alone nor continuous 808 nm laser irradiation alone does obvious harm to tumor growth; (iii) the combinatory PTT and chemotherapy could synergistically ablate cancers. These results confirm that **PATSL** is highly efficient in dual PTT and chemotherapy to cause destruction of subcutaneous solid glioma.

**FIGURE 6:**
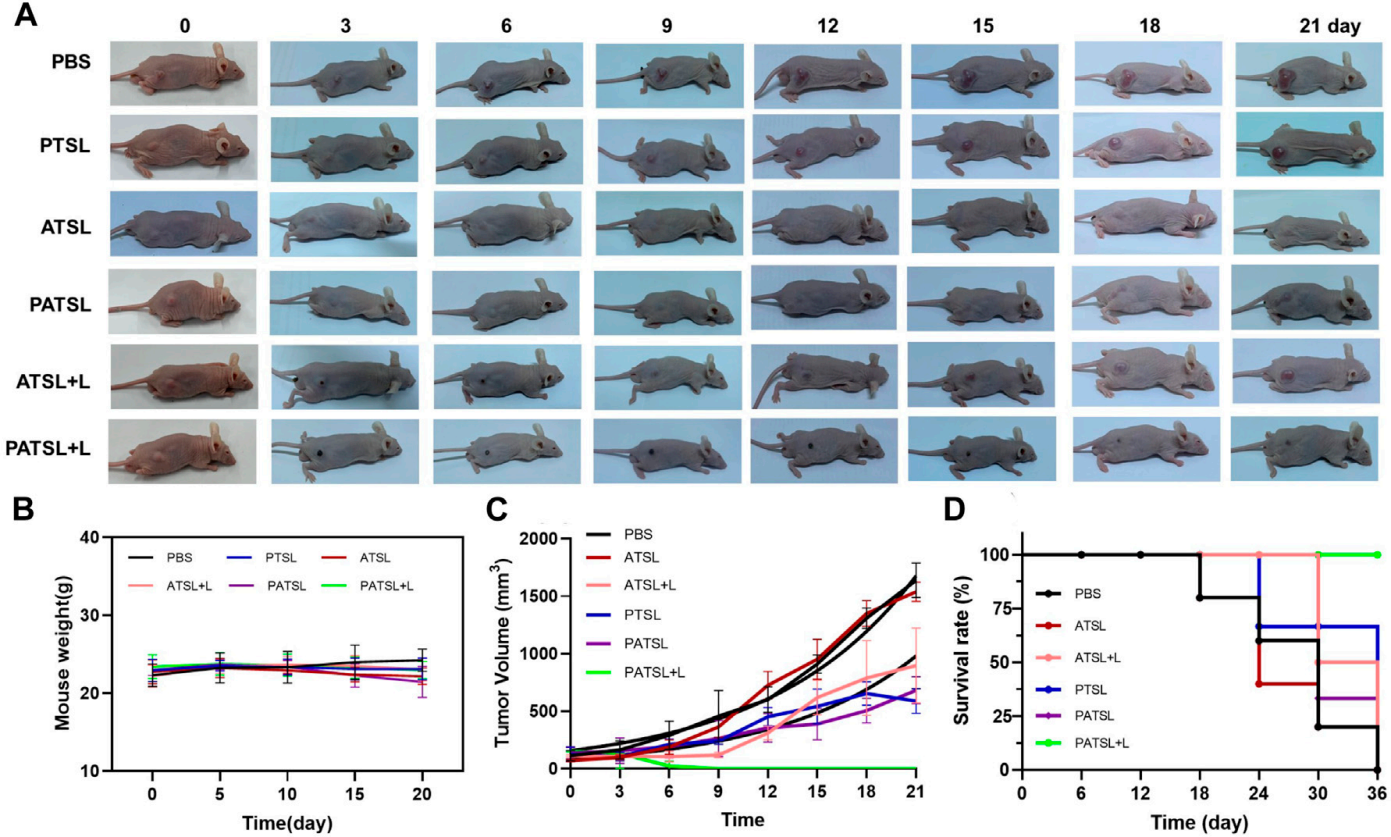
**(A)** Representative photos of mice which were bearing subcutaneous U87 glioma tumors. All the mice were divided into six groups, which include the (i) PBS group, (ii) PTSL group, (iii) ATSL group, (iv) **PATSL** group, (v) ATSL + L group, and (vi) **PATSL** + L group. **(B)** U87 tumor growth curves; **(C)** mice survival curves; **(D)** mice body weight curves.

The therapeutic effects for PTT, chemotherapy, and dual PTT and chemotherapy were evaluated in the subcutaneous glioma model *in vivo* (Figure 6). In this study, six groups of tumor-bearing mice were intratumorally treated with (i) PBS (control), (ii) **PTSL** (PTX, 1 mg kg^-1^), (iii) ATSL (**TB1**, 1 mg kg^-1^), (iv) **PATSL** (PTX + **TB1**, 2 mg kg^-1^, PTX:**TB1** = 1: 1), (v) ATSL + L (**TB1**, 1 mg kg^-1^, 808 nm laser, 0.8W/cm^2^, 5 min), and (vi) **PATSL** + L (PTX + **TB1**, 2 mg kg^-1^, PTX:**TB1** = 1: 1, 808 nm laser, 0.8W/cm^2^, 5 min). The representative pictures of mice before and after treatment in each group are illustrated in Figure 6A. As shown in Figure 6B, the mice body weight of all the six groups did not show any obvious decrease, suggesting the relatively low toxicity of the formulated nanomedicines and the corresponding treatment processes. From Figure 6C, it was found that the tumor volume in the control groups increased quickly, while the tumor volume in the **PTSL, ATSL** + L, and **PATSL** groups decreased sharply. Most importantly, some tumors in the **PATSL** + L group were completely eliminated. This suggests that synergistic combinatory PTT and chemotherapy is far better than PTT or chemotherapy alone. As shown in Figure 6D, the control groups showed a decreased survival rate since day 18. Furthermore, the singular PTT or chemotherapy groups showed better survival rate than the control groups but still suffered from the 0% survival rate after day 36. These results suggest that neither nanomedicine injection alone nor laser treatment alone can inhibit tumor growth. In contrast, for the **PATSL**+ L group, there was no tumor recurrence, demonstrating an excellent survival rate of even 100% during the experimental period.

**FIGURE 7:**
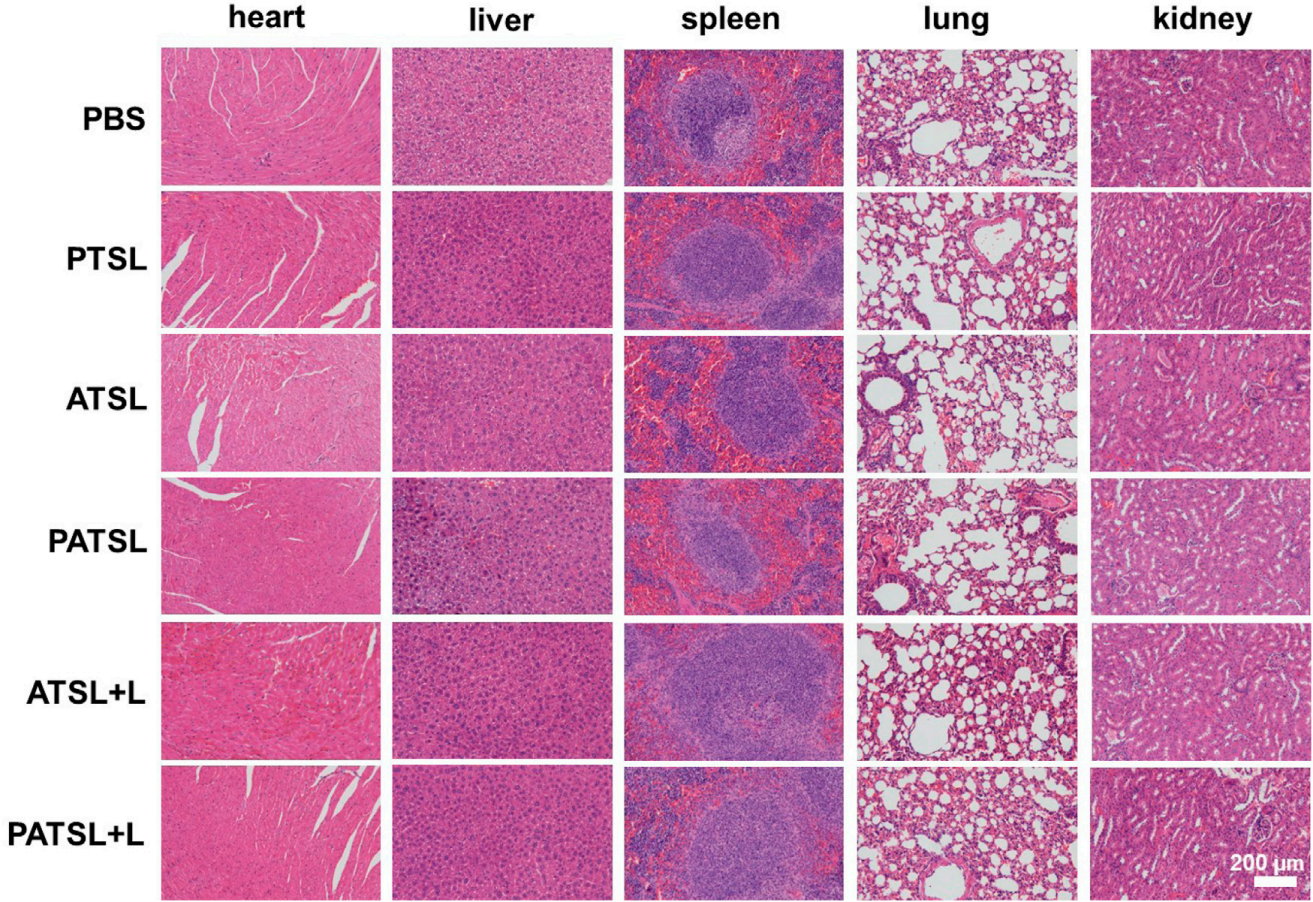
Representative hematoxylin and eosin (H&E) stained images of major organs such as the heart, liver, spleen, lung, and kidney, which were collected from the mice sacrificed after 14 days of post-injection for six groups including the (i) PBS group, (ii) PTSL group, (iii) ATSL group, (iv) **PATSL** group, (v) ATSL + L group, and (vi) **PATSL** + L group.

As shown in Figure 7, no obvious tissue damage and inflammatory lesions were visualized in major organs of mice after 14 days post-injection in different groups including the PBS group, PTSL group, ATSL group, **PATSL** group, ATSL + L group, and **PATSL** + L group, indicating their good biocompatibility.

All the above experimental results demonstrated that the “all-in-one” theranostic nanomedicine (**PATSL**) is highly efficient to ablate glioblastoma with light-activatable NIR-II fluorescence imaging-guided combinatory on-demand PTT and chemotherapy.

## 3 Conclusion

In summary, we used TSLs as drug carriers to deliver NIR-II fluorescence AIE dyes and chemotherapeutic drugs for synergistic combinatory PTT and chemotherapy of glioblastoma. The synthesized “all-in-one” nanomedicine showed stable morphology during storage, high photothermal conversion capability with an efficiency of approximately 68.6%, and good photostability during photothermal heating. The nanomedicines showed tumor imaging capability and high superiority in tumor ablation efficacy as compared to singular PTT or chemotherapy, *in vitro* and *in vivo*. Collectively, TSL-based nanomedicines containing NIR-II emissive and photothermal AIE dyes and chemotherapeutic drugs are promising candidates for “all-in-one” theranostics of glioblastoma with the treatment paradigm of light-activatable NIR-II fluorescence imaging and combinatory on-demand chemo-photothermal therapy.

## Data Availability

The original contributions presented in the study are included in the article/Supplementary Material; further inquiries can be directed to the corresponding authors.
