# Supplementary material for: Light-activatable and hyperthermia-sensitive “all-in-one” theranostics: NIR-II fluorescence imaging and chemo-photothermal therapy of subcutaneous glioblastoma by temperature-sensitive liposome-containing AIEgens and paclitaxel
Source: Front Bioeng Biotechnol. 2023 Dec 28;11:1343694. doi: 10.3389/fbioe.2023.1343694 (PMC10782224; doi:10.3389/fbioe.2023.1343694)
Supplement: Supplementary file 1 [file DataSheet1.docx]

*Supporting Information*

Light-Activatable and Hyperthermia-Sensitive “All-in-One” Theranostics: NIR-II Fluorescence Imaging and Chemo-Photothermal Therapy of Subcutaneous Glioblastoma by Temperature-Sensitive Liposomes-Containing AIEgens and Paclitaxel

Lixin Du^1^*, Pan Wang^1^, Haiyan Huang^2^, Menglong Li^2^, Shubham Roy^2^, Yinghe Zhang^2^, Bing Guo^2^*

**Materials and characterization.**

1,2-Dipalmitoyl-sn-glycero-3-phosphocholine (DPPC), 1-palmitoyl-2-hydroxy-sn-glycero-3-phosphocholine (MPPC), 1,2 distearoyl-snglycero-3-phosphoethanolamine-N-Methoxy polyethyleneglycol-2000 (DSPE-PEG2000) were purchased from Avanti Polar Lipids Inc. Paclitaxel was supplied by Shanghai Bide Pharmaceutical Technology Co., Ltd. Dulbecco's modified Eagle'smedium (DMEM) were purchased from Thermo Fisher Scientific. Fetal bovine serum (FBS) was obtained from Pan research. Cell Counting Kit-8 (CCK-8) was purchased from Zhengzhou Far East Biotechnology Co., Ltd. The NIR-II emissive AIE dye TB1 was synthesized according to literature. ^[1]^

UV−vis spectra were studied with a Shimadzu model UV-1700 spectrometer. PL spectra were tested using a Fluoromax+ fluorescence spectrometer. The NP size and its distribution were tested with laser light scattering (ZETASIZER Nano-ZS90，England) and field emission transmission electron microscopy (HT-7800, Hitachi), respectively. The 808 nm NIR laser was purchased from Changchun Lashi Optoelectronic Technology Co., Ltd.

**Synthesis of temperature-sensitive liposomes.**

***Synthesis of PATSL***. Liposomes (DPPC, MPPC and DSPE-PEG2000 in the molar ratio of 86: 10: 4) were prepared by the established thin film hydration method. Briefly, lipids, paclitaxel and TB1 were blended in chloroform (paclitaxel (PTX): TB1: lipid = 2.5:2.5:100 in weight ratio, the ratio of drug to lipid is equal to 1:20), and the resultant solution was removed under nitrogen gas at room temperature. Any residual solvent of the lipid samples was subsequently removed under vacuum for a minimum of 3 h. The dried lipid films were hydrated at 65 °C with 250 mM (NH_4_)_2_SO_4_ buffer (pH 5.4) and the suspension was extruded through a polycarbonate membrane of 200 nm using a mini-extruder (Avanti Polar Lipids, Alabaster, AL). Subsequently, the extra liposomal ammonium sulfate was replaced by PBS and the collected samples were stored in 4℃ fridge for future usage.

Empty temperature-sensitive liposome (TSL), paclitaxel-containing temperature-sensitive liposome (PTSL), and TB1-containing temperature-sensitive liposome (ATSL) were prepared by a similar approach in which PTX or TB1 was added according to the requirement.

**Size stability, and Photostability Test of PATSL.** To study PATSL photostability, the PATSL suspension at 1 mg/mL (TB1 concentration) was irradiated with a NIR laser (808 nm, 0.8 W/cm^2^) for 5 min. The UV-vis absorption spectra were recorded respectively.

**NIR laser-induced temperature increase *in vitro***

0.2 mL of PATSL (different concentration: 0, 20 ,40, 60, 80, 100 µg/mL) were added into the well of 96-well plate. NIR laser (808 nm, 0.8 W/cm^2^) was used to irradiate the samples for 5 min. The changes of temperature were obtained by infrared imaging camera (FLIR T630sc). To measure the photothermal conversion efficiency (PCE) of the PATSL (1 mg/mL), an 808 nm laser was used as the light source and the laser power was set to 0.8 W/cm^2^. The laser was switched off when the temperature reached equilibrium. These samples temperatures were naturally cooled down to ambient temperatures and recorded during the process. The PCE(η) was measured according to the following formulas:

$$\eta= \frac{hS\left( T_{max}-T_{surr} \right)-Q_{dis}}{I(1-{10}^{-A_{808}})}$$

**Where Q_dis_ represents heat dissipated of the solvent. I is the laser power (0.8 W/cm^2^), and A_808_ is the absorption intensity of the samples at 808 nm.**

**Drug encapsulation and release kinetics.**

The PTX release behavior of PATSL was studied using the dialysis method. Specifically, 2 mL sample solution (200 µg mL^−1^) was packed in a uniform pore dialysis bag (MWCO = 3500) and fastened, then immersed in 80 mL PBS solution containing 1.0 % (w/v) Tween 80. The release system was separately then placed in a 37 °C, 42 °C, 50 °C thermostatic cradle and oscillated at a frequency of 100 rpm. Then, 1 mL PBS dialysate was collected at the predetermined time point and supplemented with 1 mL fresh PBS solution. After that, constant temperature oscillation dialysis was continued, and the content of paclitaxel in each sample was calculated according to the above method for determining the paclitaxel content. The average value was obtained by measuring three times in parallel, and the time–drug cumulative release curve was plotted.

**Cell Culture and Animal Model.** Human brain glioma cell line U87 cells were supplied by American Type Culture Collection. They were cultured with Dulbecco’s Modified Eagle Medium (DMEM) media in a humidified environment with 5% CO_2_ at 37 ^o^C. The DMEM was added with 10% of fetal bovine serum and 1% of penicillin streptomycin. Before starting experiments, the cells were precultured until confluence was achieved. All animal studies were conducted according to the animal use and care regulations at Shenzhen Institutes of Advanced Technology. Balb/c nude mice (5-8 weeks old, about 20 g) were supplied by the Medical Experimental Animal Center of Guangdong. To prepare U87 tumor-bearing nudemice, U87 cells (3 × 10^6^) in PBS (100 μL) were subcutaneously injected at the nude mice. Once the tumor size increased to about 80 mm^3^, the mice were used for PA and PTT experiments.

**In vitro photothermal, chemo-, dual chemo-photothermal efficiency**

For cytotoxicity assay, 5×10^4^ of U87 per well were seeded in a 96-well plate (Costar, IL, USA) and cultured for 24 h, respectively. Then PATSL, ATSL, PTSL (15 μg/mL) were added into the 96-well plate for another 24 h. The relative cell viability was detected by standard CCK-8 assay. After removing free nanomedicines, cells were exposed to NIR laser light (808 nm, 0.8 W/cm^2^) for 5 min. The photothermal cytotoxicity was assessed by CCK-8 assay.

***In vivo* photothermal, chemo, combinatory chemo-photothermal therapy.** The balb/c nude mice bearing subcutaneous U87 tumors were separated into four groups with five animals per group as follows: (i) PBS group in which mice were only intravenously injected with PBS, (ii) PTSL group in which mice were only intratumorally injected with sample (PTX, 1 mg kg^-1^), (iii) ATSL group in which mice were only intratumorally injected with sample (**TB1**, 1 mg kg^-1^), (iv) PATSL group in which mice were only intratumorally injected with sample (PTX+TB1, 2 mg kg^-1^, PTX:**TB1** = 1: 1), (v) “ATSL+ laser” group in which mice in which mice were intratumorally injected with ATSL (2 mg **TB1**,1 mg kg^-1^), and after 5 min post-injection were irradiated with 808 nm laser (0.8 W/cm^2^, 5 min). (vi) “PATSL + laser” group in which mice were intratumorally injected with PATSL (PTX+**TB1**, 2 mg kg^-1^, PTX:**TB1** = 1: 1), and after 10 min post-injection were irradiated with 808 nm laser (0.8 W/cm^2^, 5 min). When conducting PTT, the NIR laser beam spot was focused at the center of mouse tumor. The tumor size and body weight were monitored every 3 days after PTT treatment. The tumor weight was calculated (tumor volume = length × (width)^2^/2). The measured volume value was normalized to evaluate the tumor volume. After PTT, tumors and major organs such as heart, liver, spleen, lung and kidney were collected and sectioned for H&E staining.

**H&E Staining.** H& E staining was conducted following the BBC Biochemical protocol. First, prepared cryogenic slides (8 μm) were fixed with formalin (10%) for 30 min at room temperature. Later the slides were continuously cleaned using water for 5 min and further washed with alcohol with different concentrations from 100% to 95%, and to 70% for 20 s in each concentration. The hematoxylin staining was carried out for 3 min and was washed for 1 min with water. Later the eosin staining was done in 1 min. Subsequently the slides were further washed with xylene, and mounted by Canada balsam. A Nikon Eclipse 90i microscope was used to take images.

Reference:

[1] Z. Sheng, B. Guo, D. Hu, S. Xu, W. Wu, W. Heng, L. K. Yao, J. Jiang, C. Liu, H. Zheng, B. Liu, *Adv. Mater.***2018**, *30*, 1800766.
